# The Archbishop of Canterbury Speaks to the People

**Published:** 1919-06-21

**Authors:** 


### The Archbishop of Canterbury Speaks to the People.

### THE ARCHBISHOP'S ADDRESS.

"Three hundred years ago the world's greatest poet wrote of England as

" This sceptred isle,
This fortress built by nature for herself:
This precious stone set in the silver sea."

"To-day we remember, we thank God for what our island seas have seen of heroic and tireless devotion on the part of gallant men, under conditions more perilous and more exacting than can be pictured by one in a thousand of those for whose safety they unflinchingly offered and laid down their lives.

"Never more surely than in these five incomparable years has the well-being of England depended upon her sons who were afloat. But this time it has been the well-being not of our British Isles, but of the world.

"And so we do well, King and Queen and people, to remember before God in our great Cathedral, over the graves of Nelson and Collingwood, what these men have done and given. 'These men.' Not the officers and men of the Royal Navy only, but those aboard every other craft as well. There has perhaps never been anything nobler, anything more steadily magnanimous—strange as that epithet would sound to them—than the daily, nightly courage, the cheerful resource, the sleepless vigil, say of the crew of a mine-sweeper on the North Sea, in mid-winter, or of those who manned a tug for towing war-freight from Thames to Tigris.

" Whether their fame centuries long should ring
They cared not over much,
But cared greatly to serve God and the King,
And keep the Nelson touch.
. . . . . . . . . . . .
And passed content, leaving to us the pride
Of lives obscurely great."

"It is only by slow degrees that we have come to know what things went on at sea every day, every night, during the long-drawn war. The heroisms of peril and of patience had to be firmly hidden from our eyes. We men and women at home went about our work in dusty street and mart or on rural hillside wondering, praying, sometimes listening, but without the knowledge which is now proudly ours. The tense strain, the splendid self-restraint of the men in the great ships held in leash: the monotony, unrelaxed but eager, of Harwich, or Rosyth, or Scapa Flow: the keen vigil of the dark patrol: we know them now: we could only guess before.

" And all the while we were paying bit by bit, man by man, in the cause of right, the ruthless tribute of a great war. Your lives, the pride and sunshine of countless homes, buoyantly, fearlessly offered, manfully laid down. We solemnly recall to-day that splendour of glad service. We have given of our sea forces some fifty-seven thousand lives, the very pick of our British youth and manhood, and for the nobility of their sacrifice we reverently thank God.

" Centuries hence men will tell of the exploits of Gallipoli, or Jutland, and of the skilled strategy and fertile resourcefulness of our high command. The hulk of the 'River Clyde' upon the Turkish beach, the great deed of the Marines upon the mole of Zeebrugge, will live in poetry and prose. The Army is ever foremost to testify that its task in Flanders or the East, nobly planned and stoutly done, was made possible only by the dauntless skill which guarded the waters untiringly for the ceaseless passage of armaments and men. No other record like to this stands in the annals of the seas.

"And, brothers, we all know it, it has been worth while. It is worth while. We have lived through large days, large doings, at a great juncture in the history of mankind. Through the testing fires we have striven to reach the height of an ennobling trust. And we are not afraid, as we ponder that sacrifice and all that it means, we are not afraid, weak and wayward though we be, to link it with our prayers for the bettering of the world, for the coming o' the Kingdom of the Prince of Peace."

